# Genomes of two indigenous clams *Anomalocardia flexuosa* (Linnaeus, 1767) and *Meretrix petechialis* (Lamarck, 1818)

**DOI:** 10.1038/s41597-025-04748-9

**Published:** 2025-03-08

**Authors:** Sean Tsz Sum Law, Wenyan Nong, Ming Fung Franco Au, Leni Hiu Tong Cheung, Cheryl Wood Yee Shum, Shing Yip Lee, Siu Gin Cheung, Jerome Ho Lam Hui

**Affiliations:** 1 School of Life Sciences, Simon F.S. Li Marine Science Laboratory, Institute of Environment, Energy and Sustainability, State Key Laboratory of Agrobiotechnology, The Chinese University of Hong Kong, Hong Kong SAR, China; 2 Simon F.S. Li Marine Science Laboratory, School of Life Sciences, The Chinese University of Hong Kong, Hong Kong SAR, China; 3 Australian Rivers Institute, Griffith University Gold Coast campus, Southport, Qld 4222 Australia; 4 Department of Chemistry, State Key Laboratory of Marine Pollution, City University of Hong Kong, Hong Kong SAR, China

**Keywords:** Conservation biology, Zoology

## Abstract

Clam digging has a long history in Hong Kong, but unregulated clam digging activities depletes clam populations and threatens the ecosystem. Population genomics is useful to unravel the connectivity of clams at different geographical locations and to provide necessary conservation measures; and yet, only limited number of clams in Hong Kong have genomic resources. Here, we present chromosomal-level genome assemblies for two clams commonly found in Hong Kong, *Anomalocardia flexuosa* and *Meretrix petechialis*, using a combination of PacBio HiFi and Omni-C reads. For *A. flexuosa*, we assembled the genome into 19 pseudochromosomes with a genome size of 1.09 Gb (scaffold N50 = 58.5 Mb), and BUSCO scores of 94.4%. A total of 20,881 gene models were also predicted using the transcriptomes generated in this study. For *M. petechialis*, the genome was mainly assembled into 19 pseudochromosomes with a genome size of 1.04 Gb (scaffold N50 = 53.5 Mb), and BUSCO scores of 95.7%. A total of 20,084 gene models were also predicted using the transcriptomes generated in this study. The two new genomic resources established in this study will be useful for further study of biology, ecology, and evolution of clams, as well as setting up a foundation for evidence-informed decision making in conservation measures and implementation.

## Background & Summary

Clams refer to the common name for several kinds of bivalve molluscs. The Veneridae family contains more than 700 described living species of bivalves or clams, and most of them are edible and exploited as food in different cultures around the world, including America, Asia and Europe (Huber, 2010)^1^. Clam digging activities, which refer to harvesting clams from below the surface of tidal sand or mud flats, also has long history in many places including Hong Kong. In the last century, clam digging in Hong Kong were mainly confined to villagers or recreational collection using hand tools on beaches during low tides for consumption or as a source of income. Nevertheless, clam digging activities have grown increasingly popular in recent years which threatens the clam populations and disturbs benthic biodiversity in some areas (Griffiths *et al*.; So *et al*.)^2,3^. Unlike many other places where sustainable clam digging practices, such as limiting the number of clams taken and/or temporary closure of clamming sites, Hong Kong does not have her own practices in the meantime due to the lack of information on the population structure of clams. As of to date, 12 genome assemblies in the Veneridae are available in NCBI (8 November 2024), including five chromosome-level genomes in the genera *Mercenaria*, *Mysia*, *Ruditapes* and *Venus*. Among the common clams that can be found in Hong Kong, such as that of *Anomalocardia* and *Meretrix* species which are the two frequently collected genera by local clam-diggers (So *et al*.)^3^, genomic resources are currently lacking which hinders our understanding of their connectivity at different geographical locations.

Here, utilizing PacBio HiFi long reads and Omni-C sequencing data, we present two chromosomal-level genomes of common clams in Hong Kong, *Anomalocardia flexuosa* and *Meretrix petechialis*. Together with transcriptome data from various tissues, we produce high-quality predicted gene models for the two clam species. These genome assemblies and transcriptome data provide valuable genomic resources for the understanding of genetic diversity and connectivity for future population genomics research in view of conserving local clam species and assessing the sustainability of clam digging activities.

## Methods

### Sample collection and high molecular weight DNA extraction

**Fig. 1:**
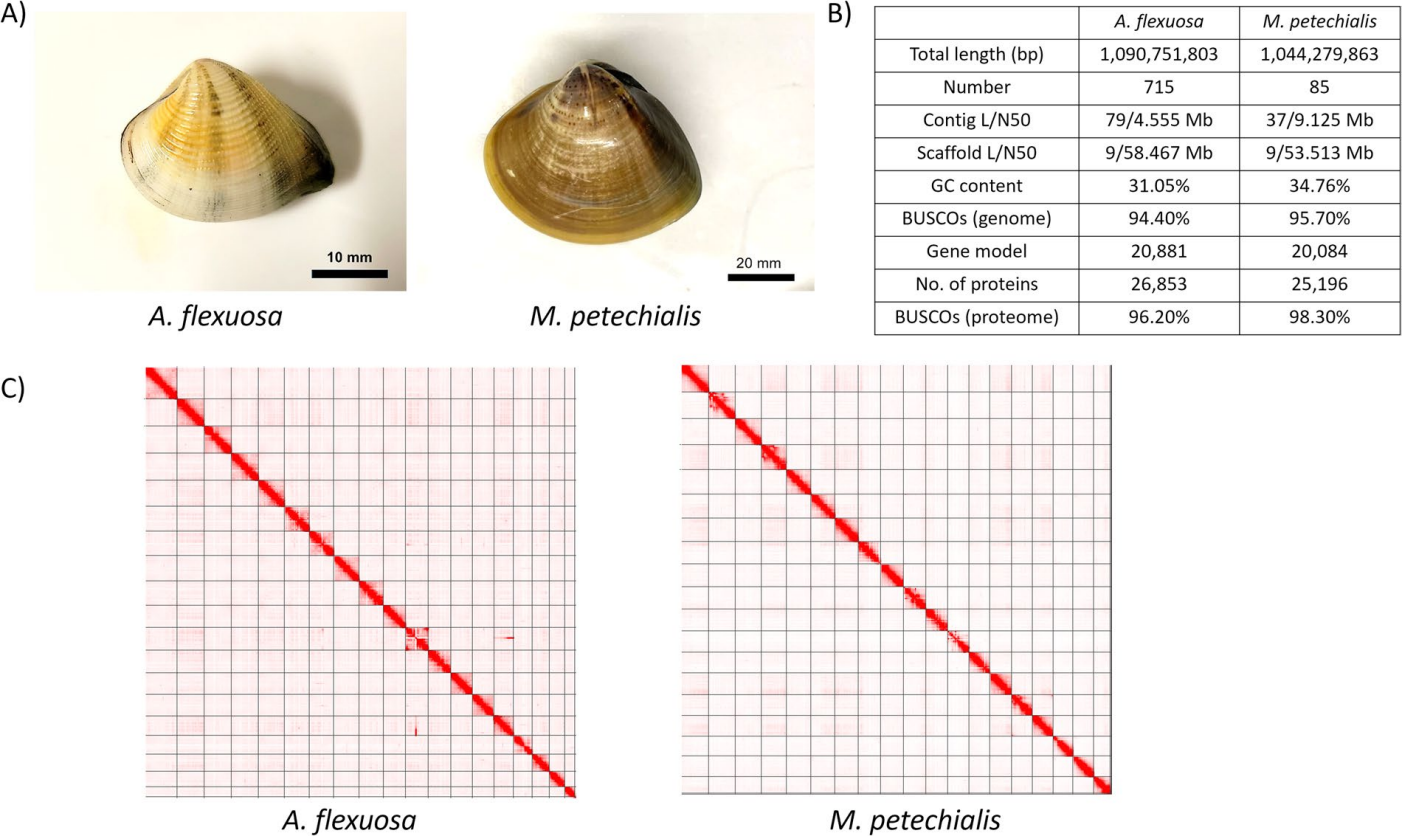
(**A**) Pictures of *A. flexuosa* (left) and *M. petechialis* (right); (**B**) Statistics of the genome assembly generated in this study; (**C**) Hi-C contact map of the assembly *A. flexuosa* (left) and *M. petechialis* (right); (**D**).

*A. flexuosa* and *M. petechialis* samples were collected in Shui Hau, Lantau Island, Hong Kong (22°13′14.2″N 113°55′09.0″E) on 6^th^ July, 2023 and Yi O, Lantau Island, Hong Kong (22°13′58.4″N 113°51′02.0″E), on 28^th^ August, 2022, respectively (Fig. 1A). Approximately 300 mg adductor muscle was used for high molecular weight (HMW) DNA extraction for both *A. flexuosa* and *M. petechialis*. For *A. flexuosa*, the tissue was first ground into powder with liquid nitrogen, from which HMW DNA was isolated by NucleoBond HMW DNA kit (Macherey-Nagel), following the manufacturer’s protocol. For *M. petechialis*, HMW DNA was extracted using MagAttract HMW DNA Kit (Qiagen), following the manufacturer’s instructions. The DNA samples were eluted with 120 µL of elution buffer (PacBio Cat. No. 101-633-500) and were subjected to quality check by the Qubit® Fluorometer, NanoDrop One Spectrophotometer, and overnight pulse-field gel electrophoresis.

### PacBio library preparation and long-read sequencing

Prior to library preparation, approximately 5 µg of HMW DNA isolated from *A. flexuosa* and *M. petechialis* in 120 µL of elution buffer were transferred to a g-tube (Covaris Cat. No. 520079) for DNA shearing with 6 passes of centrifugation at 1,990 × *g* for 2 min. The fragment size of sheared DNA samples was assessed with overnight pulse-field gel electrophoresis. A SMRTbell library was constructed for both samples using the SMRTbell® prep kit 3.0 (PacBio Cat. No. 102-141-700), following the manufacturer’s instructions. Qubit® Fluorometer and overnight pulse-field gel electrophoresis were used to examine the quantity and quality of the SMRTbell libraries. Subsequently, the Sequel®II binding kit 3.2 (PacBio Cat. No. 102-194-100) was used for the final library preparation. Briefly, 3 µL of the SMRTbell library was mixed with 1.5 µL annealing buffer and 1.5 µL Sequel II Primer 3.2, and further incubated for 15 minutes in room temperature. Subsequently, a dilution step of Sequel II DNA polymerase 2.2 was carried out according to the manufacturer’s instructions, where 6 µL diluted polymerase was added to the SMRTbell mixture and incubated for 15 minutes in room temperature for polymerase binding, and followed by a purification step using SMRTbell® clean-up beads. The polymerase-bound complexes were eluted with 50 µL Sequel II Loading Buffer 3.2, which were then mixed with an addition of 67 µL Sequel II Loading Buffer 3.2 and 3 µL diluted internal DNA control to prepare a final loading library of 120 µL in volume. All mixing procedures during the SMRTbell library preparation and the final library preparation were performed with 200 µL wide bore tips (Rannin Cat No. 30389188) in 2 mL DNA LoBind® Tubes (Eppendorf Cat No. 022431048). 115 µL of the two final libraries were loaded at an on-plate concentration of 90 pM with diffusion loading mode, respectively. The sequencing was performed on the PacBio Sequel IIe system using circular consensus sequencing (CCS) sequencing mode for a 30-hour movie with 2-hour pre-extension to generate HiFi reads for each sample. One SMRT cell was used for sequencing for *A. flexuosa* and *M. petechialis*, respectively. Finally, 21.83 Gb and 30.58 Gb of Hifi reads were obtained for *A. flexuosa* and *M. petechialis* with average lengths of 8,017 bp and 10,729 bp and data coverages of 20X and 29X, respectively (Table 1).

**Table 1:** Genome and transcriptome sequencing data.

Species	Samples	Reads	Bases	Accesion number
Genome sequencing data				
*Anomalocardia_flexuosa*	Afle_HiFi	2,666,215	21,375,799,631	SRR28740828
*Anomalocardia_flexuosa*	Afle_omnic	402,870,354	60,430,553,100	SRR28728919
*Meretrix_petechialis*	Mpet_HiFi	2,850,426	30,583,425,339	SRR28712952
*Meretrix_petechialis*	Mpet_omnic	377,451,754	56,617,763,100	SRR28712977
Transcriptome sequencing data				
*Anomalocardia_flexuosa*	Af_Dg (Digestive gland)	44,264,938	6,639,733,793	SAMN41013774
*Anomalocardia_flexuosa*	Af_Gl (Gill)	43,570,390	6,535,551,419	SAMN41013775
*Anomalocardia_flexuosa*	Af_Gn (Gonad)	42,937,050	6,440,550,167	SAMN41013776
*Anomalocardia_flexuosa*	Af_Mc (foot and adductor muscle)	39,583,428	5,937,506,340	SAMN41013777
*Anomalocardia_flexuosa*	Af_Mt (Mantle)	41,021,228	6,153,176,405	SAMN41013778
*Meretrix_petechialis*	Mp9YO_Dg (Digestive gland)	37,997,880	5,699,679,344	SAMN41013738
*Meretrix_petechialis*	Mp9YO_Ft (foot muscle)	38,741,520	5,811,225,289	SAMN41013739
*Meretrix_petechialis*	Mp9YO_Gl (Gill)	39,108,764	5,866,311,436	SAMN41013740
*Meretrix_petechialis*	Mp9YO_Gn (Gonad)	38,134,110	5,720,113,520	SAMN41013741

### Omni-C library preparation and sequencing

An Omni-C library was prepared for *A. flexuosa* and *M. petechialis*, respectively, using the Dovetail® Omni-C® Library Preparation Kit (Dovetail Cat. No. 21005), following the manufacturer’s instructions. Approximately 50 mg of flash-freezing powered tissue was used for crosslinking with the addition of formaldehyde in 1 mL 1X PBS for each sample, followed by nuclease digestion. The lysate samples were assessed by Qubit® Fluorometer and TapeStation D5000 ScreenTape and were proceeded with the library preparation protocol. After the final quality check with Qubit® Fluorometer and TapeStation D5000 ScreenTape, the Omni-C libraries were sent to Novogene Co. Ltd for sequencing on an Illumina HiSeq-PE150 platform, from which 60.4 Gb and 56.6 Gb Omni-C data were generated for *A. flexuosa* and *M. petechialis*, respectively (Table 1).

### Transcriptome sequencing

Total RNA was isolated from various tissues including foot and adductor muscle, mantle, digestive gland, gill and gonad for *A. flexuosa* and foot, digestive gland, gill and gonad for *M. petechialis*, using the TRIzol™ Reagent (Invitrogen Cat No. 15596018), following the manufacturer’s protocol respectively. The RNA samples were subjected to quality control using NanoDrop One Spectrophotometer, and gel electrophoresis. The qualified samples were sent to Novogene Co. Ltd for polyA selected RNA sequencing library construction and 150 bp paired-end sequencing. A total of 31.7 Gb and 23.1 Gb transcriptome data were obtained from different tissue types of *A. flexuosa* and *M. petechialis*, respectively (Table 1).

### Genome assembly and Gene model prediction

*De novo* genome assemblies of *A. flexuosa* and *M. petechialis* were first proceeded with Hifiasm (Cheng *et al*.)^4^ and then were processed with searching against the NT database with BLAST to remove possible contaminations using BlobTools (v1.1.1) (Laetsch & Blaxter)^5^. Subsequently, haplotypic duplications were removed according to the depth of HiFi reads using “purge_dups” (Guan *et al*.)^6^. Proximity ligation data from Omni-C were used to scaffold the assembly using YaHS (Zhou *et al*.)^7^ and manual checking using Juicebox (v1.1)^8^. The genomes were soft-masked by redmask (v0.0.2) (https://github.com/nextgenusfs/redmask) (Girgis *et al*.)^9^. The final genome assemblies of *A. flexuosa* and *M. petechialis* were 1.09 Gb and 1.04 Gb in size with 95.43% and 99.27% of the sequenced anchored into 19 chromosomes, and 391 and 195 gaps, respectively, which correspond to the kartotype (2n = 38) of *Anomalocardia* and *Meretrix* species (Fig. 1B-C; Tables 2, 3) (Lavander *et al*.; Park *et al*.)^10,11^. Both *A. flexuosa* and *M. petechialis* genomes were not only of high continuity, with scaffold N50 of 58.5 Mb and 53.5 Mb in 9 scaffolds, but also of high completeness after being assessed with Benchmarking Universal Single-Copy Orthologs (BUSCO, v5.5.0) using the “metazo_odb10” dataset (Manni *et al*.)^12^, which resulted in BUSCO scores of 94.4% (Complete and single-copy BUSCOs (S): 92.8%, Complete and duplicated BUSCOs (D): 1.6%, Fragmented BUSCOs (F): 3.7%, Missing BUSCOs (M): 1.9%) and 95.7% (S:93.2%, D:2.5%, F:2.7%, M:1.6%), respectively (Fig. 1B; Table 2).

**Table 2:** Genome statistics.

Species	Anomalocardia flexuosa	Meretrix petechialis
Total length (bp)	1,090,751,803	1,044,147,259
Number	715	85
Mean length (bp)	1,525,527	12,284,085
Longest	76,979,603	65,874,028
Shortest	1,000	1,000
N count	78,200	39,200
Gaps	391	195
N50	58,466,825	53,512,864
N50n	9	9
N70	52,832,318	51,039,052
N70n	13	13
N90	37,743,970	49,958,606
N90n	18	17
HiFi reads coverage (X)	20	29
Reads	2,666,215	2,850,426
Bases	21,375,799,631	30,583,425,339
HiFi reads average length (bp)	8,017	10,729
BUSCOs (genome)	C:94.4%[S:92.8%,D:1.6%],F:3.7%,M:1.9%,n:954	C:95.7%[S:93.2%,D:2.5%],F:2.7%,M:1.6%,n:954
No. of proteins	26,853	25,196
BUSCOs (proteome)	C:96.2%[S:82.0%,D:14.2%],F:0.4%,M:3.4%,n:954	C:98.3%[S:81.7%,D:16.6%],F:0.1%,M:1.6%,n:954

**Table 3:** Pseudochromosome information.

Chr no.	Scaffold id	Scaffold length (bp)	Cumulative % of the whole genome
*Anomalocardia flexuosa*			
1	scaffold_1	76,979,603	0.07
2	scaffold_2	66,208,884	0.13
3	scaffold_3	65,610,396	0.19
4	scaffold_4	64,669,910	0.25
5	scaffold_5	64,436,222	0.31
6	scaffold_6	59,912,551	0.36
7	scaffold_7	59,882,163	0.42
8	scaffold_8	59,794,789	0.47
9	scaffold_9	58,466,825	0.53
10	scaffold_10	55,963,395	0.58
11	scaffold_11	53,985,618	0.63
12	scaffold_12	53,674,951	0.68
13	scaffold_13	52,832,318	0.73
14	scaffold_14	52,636,158	0.77
15	scaffold_15	48,317,914	0.82
16	scaffold_16	45,620,769	0.86
17	scaffold_17	41,163,429	0.90
18	scaffold_18	37,743,970	0.93
19	scaffold_19	22,996,108	0.95
*Meretrix petechialis*			
1	scaffold_1	65,874,028	0.06
2	scaffold_2	64,475,212	0.12
3	scaffold_3	62,842,314	0.19
4	scaffold_4	60,700,655	0.24
5	scaffold_5	59,338,163	0.30
6	scaffold_6	58,351,309	0.36
7	scaffold_7	56,358,383	0.41
8	scaffold_8	55,055,675	0.46
9	scaffold_9	53,512,864	0.51
10	scaffold_10	53,341,073	0.56
11	scaffold_11	53,188,068	0.62
12	scaffold_12	51,880,032	0.67
13	scaffold_13	51,039,052	0.71
14	scaffold_14	50,896,477	0.76
15	scaffold_15	50,371,808	0.81
16	scaffold_16	49,989,395	0.86
17	scaffold_17	49,958,606	0.91
18	scaffold_18	49,380,020	0.95
19	scaffold_19	39,964,719	0.99

For gene model prediction, RNA sequencing data were first processed using Trimmomatic (v0.39) (Bolger, Lohse & Usadel)^13^ with parameters “TruSeq. 3-PE.fa:2:30:10 SLIDINGWINDOW:4:5 LEADING:5 TRAILING:5 MINLEN:25” and kraken2 (v2. 0.8 with kraken2 database k2_standard_20210517)^14^ to remove the low quality and contaminated reads, and then aligned to the repeat soft-masked genome using Hisat2^15^ to generate the bam file. A total of 389,399 Mollusca reference protein sequences were downloaded from NCBI on 25 March 2024 as protein hits, along with the RNA bam file, to perform genome annotation using Braker (v3.0.8)^16^ with default parameters. Briefly, RNA-Seq and protein hints were used to train GeneMark-ETP, from which the genes with high extrinsic evidence support were then used to train on AUGUSTUS. The predictions from AUGUSTUS and GeneMark-ETP were combined using TSEBRA to generate the final annotation files.

For *A. flexuosa*, the transcriptome assembled by stringtie (v2.2.1)^17^ contained 53,191 transcripts in a total of 131,817,025 bp with an average length of 2,478 bp and an N50 length of 4,030 bp. The completeness of the transcriptome was also assessed using the BUSCO “metazo_odb10” dataset (Manni *et al*.)^12^, reporting a BUSCO score of 98.1%. The assembled transcriptome was then used for gene model prediction. These data collectively generated 20,881 gene models, comprising 26,853 predicted protein-coding genes with average lengths of 580 amino acids (Fig. 1B; Table 2). The completeness of proteomes were also evaluated with BUSCO “metazo_odb10” dataset (Manni *et al*.)^12^, reporting BUSCO scores of 96.2% (Fig. 1B; Table 2). Protein-coding genes were mapped to the nr and swissprot databases using Diamond (v2.0.7)^18^ with the parameter ‘--evalue 1e-3--outfmt 6’ for functional annotation, and 97.56% of the protein-coding genes could be mapped to the database. The transcriptome data was mapped to gene modes using hisat2^15^ with default parameters, 96.82% of genes are expressed in the transcriptome samples.

For *M. petechialis*, the transcriptome assembled by stringtie (v2.2.1)^17^ contained 28,098 transcripts in a total of 83,316,136 bp with an average length of 2,965 bp and an N50 length of 4,271 bp. The completeness of the transcriptome was also assessed using the BUSCO “metazo_odb10” dataset (Manni *et al*.)^12^, reporting a BUSCO score of 97.6%. The assembled transcriptome was then used for gene model prediction. These data collectively generated 20,084 gene models, comprising 25,196 predicted protein-coding genes with average lengths of 607 amino acids (Fig. 1B; Table 2). The completeness of proteomes were also evaluated with BUSCO “metazo_odb10” dataset (Manni *et al*.)^12^, reporting BUSCO scores of 98.3% (Fig. 1B; Table 2). Protein-coding genes were mapped to the nr and swissprot databases using Diamond (v2.0.7)^18^ with the parameter ‘--evalue 1e-3--outfmt 6’ for functional annotation, and 96.72% of the protein-coding genes could be mapped to the database. The transcriptome data was mapped to gene modes using hisat2^15^ with default parameters, 95.96% of genes are expressed in the transcriptome samples.

### Repetitive elements annotation

**Fig. 2:**
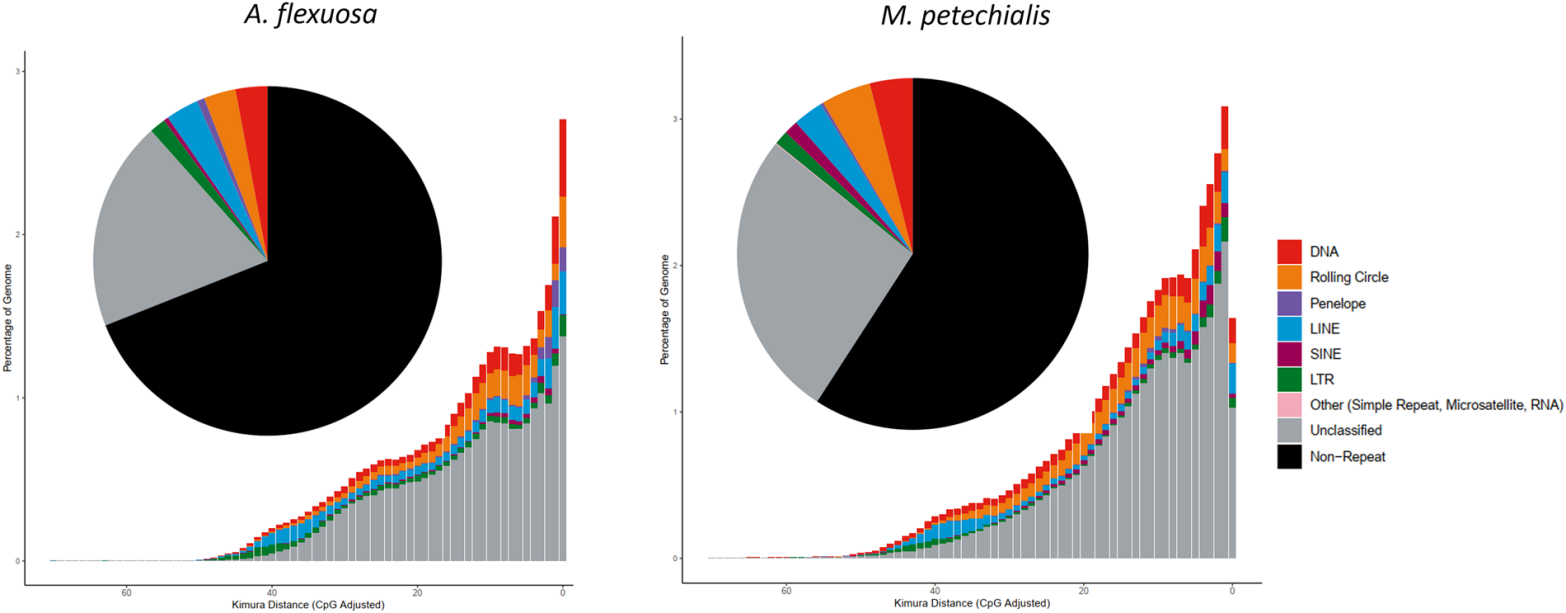
Pie chart and repeat landscape plot of repetitive elements of *A. flexuosa* (left) and *M. petechialis* (right).

Transposable elements (TEs) of the two genome assemblies were annotated as previously described (Baril *et al*.)^19^ using the automated Earl Grey TE annotation pipeline (version 1.2, https://github.com/TobyBaril/EarlGrey) with “-r eukarya” to search the initial mask of known elements and other default parameters. Briefly, this pipeline first identified known TEs from Dfam with RBRM (release 3.2) and RepBase (v20181026). *De novo* TEs were then identified, and consensus boundaries were extended using an automated “BLAST, Extract, Extend’ process with 5 iterations and 1,000 flanking bases added in each round. Redundant sequences were removed from the consensus library before the genome assembly was annotated with the combined known and *de novo* TE libraries. Overlap and defragment annotations were removed prior to final TE quantification. A total of 338.3 Mb and 427.2 Mb of repeat contents were annotated from the genomes of *A. flexuosa* and *M. petechialis*, which account for 31.02% and 40.92% of the assembly, respectively (Fig. 2; Table 4). Of the classified TEs, LINE, DNA, and Rolling Circle contribute to the major proportions (Fig. 2), which are listed in Table 4. Repeat landscape plots of both *A. flexuosa* and *M. petechialis* revealed substantial increase of activities of LINE, DNA, and Rolling Circle and LTR, although a sharp recent decrease in repetitive element activities was observed in *M. petechialis* (Fig. 2). 19.36% and 26.65% of the repeat content were marked as unclassified for *A. flexuosa* and *M. petechialis* respectively. Therefore, we also run the Extensive de novo TE Annotator (EDTA)^20^ with parameters “--species others--threads 32--cds cds.fa--force 1--anno 1 “ for comparison. Despite fewer repetitive content was annotated by EDTA than earlGrey method in *A. flexuosa* (22.00%) and *M. petechialis* (31.15%), TIR still accounts for nearly half of the annotated repetitive elements annotated by either method (Tables 5, 6). Previous studies also identified the expansion and diversification of transposon elements in bivalve genomes (Farhat *et al*.; Martelossi *et al*.)^21,22^. Therefore, the genomes of *A. flexuosa* and *M. petechialis* may serve as valuable resources for further studies of genome evolution in Veneridae.

**Table 4:** EarlGrey repeat content summary.

Classification	Coverage (bp)	Count	Proportion (%)	No. of distinct classifications
*A. flexuosa*				
DNA	32,355,348	49,773	2.97	6,979
LINE	33,557,383	42,257	3.08	6,509
LTR	16,669,352	16,758	1.53	4,072
Other (Simple Repeat, Microsatellite, RNA)	162,593	439	0.01	329
Penelope	7,635,347	5,280	0.70	2,121
Rolling Circle	32,188,869	35,849	2.95	3,391
SINE	4,543,005	11,316	0.42	1,466
Unclassified	211,213,474	381,513	19.36	9,250
SUM	338,325,371	543,185	31.02	34,117
*M. petechialis*				
DNA	41,261,575	67,500	3.95	7,216
LINE	29,088,046	35,521	2.79	5,620
LTR	13,628,534	12,061	1.31	4,252
Other (Simple Repeat, Microsatellite, RNA)	975,819	1,065	0.09	728
Penelope	2,806,372	4,369	0.27	2,208
Rolling Circle	47,821,213	64,168	4.58	3,373
SINE	13,352,869	53,759	1.28	1,228
Unclassified	278,284,523	493,996	26.65	8,896
SUM	427,218,951	732,439	40.92	33,521

**Table 5:** EDTA repeat content summary.

Class	Sub class	A. flexuosa			M. petechialis		
		Count	Coverage (bp)	Proportion (%)	Count	Coverage (bp)	Proportion (%)
LINE	CR1	3,705	1,587,891	0.15	8,602	2,786,199	0.27
	I	1,661	1,280,085	0.12	9,909	4,497,473	0.43
	Jockey	353	226,456	0.02	/	/	/
	L1	5,894	3,520,180	0.32	5,348	2,976,001	0.29
	L2	7,109	3,632,478	0.33	5,551	2,659,894	0.25
	Proto2	2,001	718,443	0.07	735	557,396	0.05
	R2	83	41,096	0.00	415	261,746	0.03
	RTE	16,403	9,137,736	0.84	16,934	8,020,547	0.77
	unknown	3,347	746,822	0.07	515	358,762	0.03
LTR	Copia	75	50,987	0.00	206	141,833	0.01
	Gypsy	35,955	22,111,187	2.03	70,827	39,102,451	3.75
	unknown	145,153	46,830,883	4.29	180,620	47,254,644	4.53
SINE	5S	2,179	423,609	0.04	215	62,099	0.01
	B2	/	/	/	1,147	194,260	0.02
	B4	/	/	/	580	88,098	0.01
	MIR	/	/	/	2,996	421,895	0.04
	tRNA	40	3,846	0.00	473	95,039	0.01
TIR	CACTA	121,235	32,582,612	2.99	199,577	47,915,739	4.59
	Mutator	106,489	31,931,868	2.93	184,499	48,139,374	4.61
	PIF_Harbinger	49,470	16,811,316	1.54	115,421	32,726,416	3.13
	Tc1_Mariner	6,802	5,909,616	0.54	10,153	3,082,456	0.30
	hAT	48,588	17,270,407	1.58	107,465	31,909,218	3.06
nonLTR	Penelope	4,175	4,629,802	0.42	5,522	2,074,138	0.20
nonTIR	helitron	91,445	23,765,704	2.18	154,749	35,312,100	3.38
repeat_region		64,118	16,752,765	1.54	58,890	14,389,456	1.38
Total		716,280	239,965,789	22.00	1,141,349	325,027,234	31.15

**Table 6:** Comparison of repeat annotation from EarlGrey and EDTA.

Classification	*A. flexuosa*		*M. petechialis*	
	earlGrey Proportion (%)	EDTA Proportion (%)	earlGrey Proportion (%)	EDTA Proportion (%)
DNA	2.97%	/	3.95%	/
LINE	3.08%	1.92%	2.79%	2.12%
LTR	1.53%	6.32%	1.31%	8.29%
Other	0.01%	1.54%	0.09%	1.38%
Penelope	0.70%	0.42%	0.27%	0.20%
Rolling Circle	2.95%	/	4.58%	/
SINE	0.42%	0.04%	1.28%	0.09%
Unclassified	19.36%	/	26.65%	/
TIR	/	9.58%	/	15.69%
helitron	/	2.18%	/	3.38%
SUM	31.02%	22.00%	40.92%	31.15%

### Syntenic analyses

**Fig. 3:**
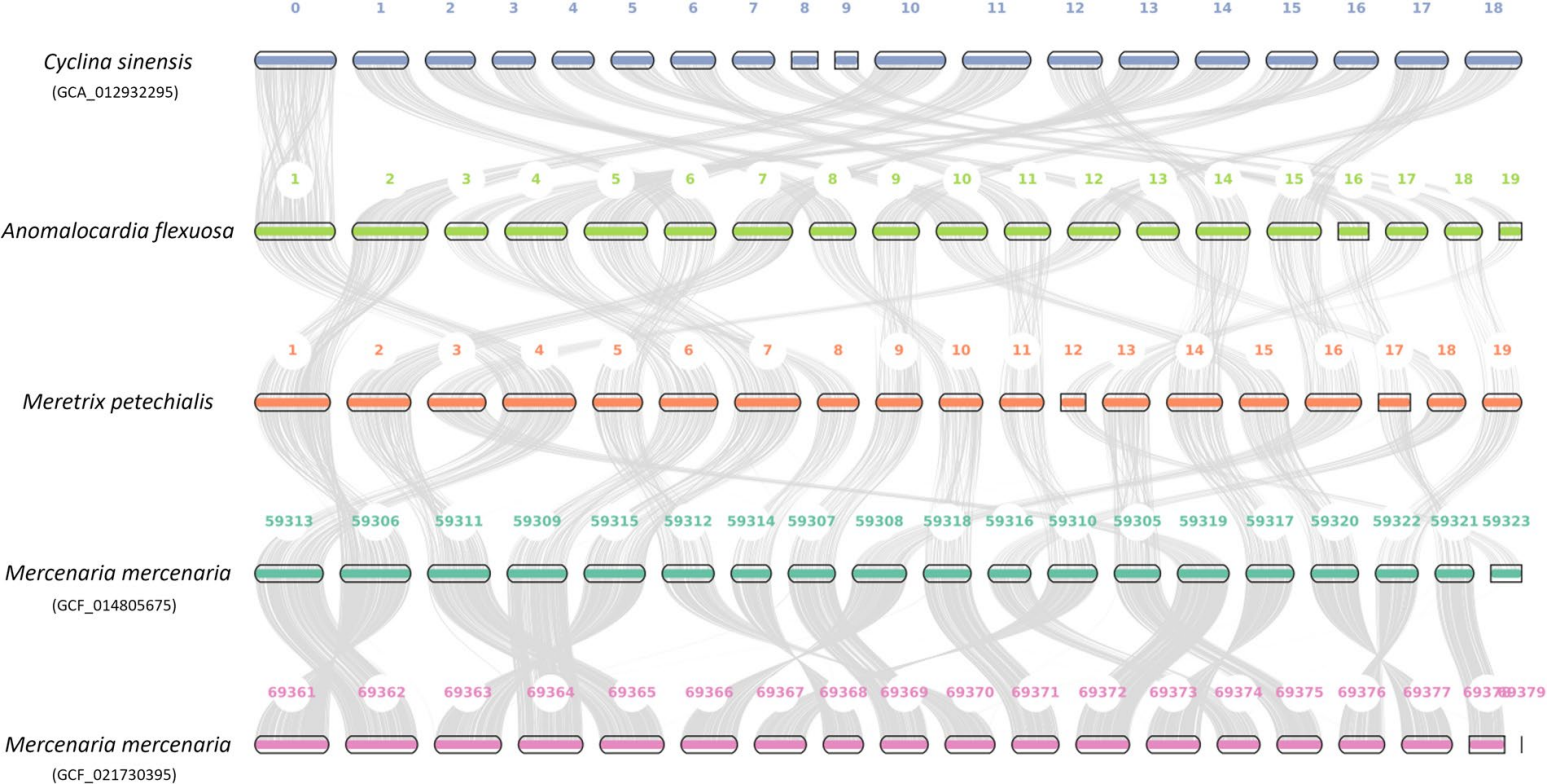
Macrosynteny plot of the 19 pseudochromosomes between *Cyclina sinensis*, *A. flexuosa*, *M. petechialis* and *Mercenaria mercenaria* (Mmer).

Macrosynteny analysis revealed a 1-to-1 pair relationship between the 19 pseudochromsomes of *Cyclina sinensis*^23^, *A. flexuosa*, *M. petechialis* and *Mercenaria mercenaria*^21^ using JCVI^24^ (Fig. 3), showing a conserved chromosome architecture between the four species, and the same chromosome number as in other Veneridae genomes. The full genome details downloaded from NCBI on 24 October 2024 are shown in Table 7.

**Table 7:** Comparison of the Venerida genomes.

Assembly Accession	Organism Name	Family	Genus	Organism Taxonomic ID	Assembly Stats Total Sequence Length	Assembly Stats Total Number of Chromosomes	Assembly Level	Assembly Stats Scaffold N50	Annotation Count Gene Total
**JBCAUM000000000**	***Anomalocardia flexuosa***	**Veneridae**	**Anomalocardia**	**3139943**	**1,090,751,803**	**19**	**Chromosome**	**58,466,825**	**20,881**
GCF_021730395.1	*Mercenaria mercenaria*	Veneridae	Mercenaria	6596	1,858,199,728	19	Chromosome	82,914,371	45,375
GCF_014805675.1	*Mercenaria mercenaria*	Veneridae	Mercenaria	6596	1,777,616,429	19	Chromosome	91,379,220	43,960
**JBCJFF000000000**	***Meretrix petechialis***	**Veneridae**	**Meretrix**	**311198**	**1,044,147,259**	**19**	**Chromosome**	**53,512,864**	**20,084**
GCA_964106805.1	*Mysia undata*	Veneridae	Mysia	1920014	1,613,701,415	19	Chromosome	85,061,451	/
GCA_009026015.1	*Ruditapes philippinarum*	Veneridae	Ruditapes	129788	1,123,164,463	19	Chromosome	345,005	/
GCA_964200665.1	*Venus verrucosa*	Veneridae	Venus	55715	2,087,848,406	19	Chromosome	108,501,084	/
GCF_026571515.1	*Ruditapes philippinarum*	Veneridae	Ruditapes	129788	1,408,116,862	/	Contig	183,074	48,037
GCA_012932295.1	*Cyclina sinensis*	Veneridae	Cyclina	120566	903,119,975	/	Scaffold	46,470,132	/
GCA_964106865.1	*Mysia undata*	Veneridae	Mysia	1920014	1,617,548,918	/	Scaffold	86,720,194	/
GCA_022818135.1	*Saxidomus purpurata*	Veneridae	Saxidomus	311201	1,161,000,147	/	Scaffold	52,225,674	/
GCA_032359765.1	*Saxidomus gigantea*	Veneridae	Saxidomus	410349	921,158,569	/	Scaffold	3,721	/
GCA_041429985.1	*Tivela stultorum*	Veneridae	Tivela	345375	763,638,067	/	Scaffold	38,630,951	/
GCA_041429975.1	*Tivela stultorum*	Veneridae	Tivela	345375	738,096,130	/	Scaffold	40,863,990	/

## Data Records

The genome assemblies are in GenBank under accessions JBCAUM000000000^25^ (*A. flexuosa*) and JBCJFF000000000^26^ (*M. petechialis*). The raw reads generated in this study, including Transcriptome, Omni-C and PacBio HiFi data, have been deposited in the NCBI database under the SRA accession number SRP502500^27^ and SRP502172^28^ for *A. flexuosa* and *M. petechialis*, respectively. The genome, genome and repeat annotation files have been deposited and are publicly available in Figshare^29^ and CUHK Research Data Repository^30^.

## Technical Validation

**Fig. 4:**
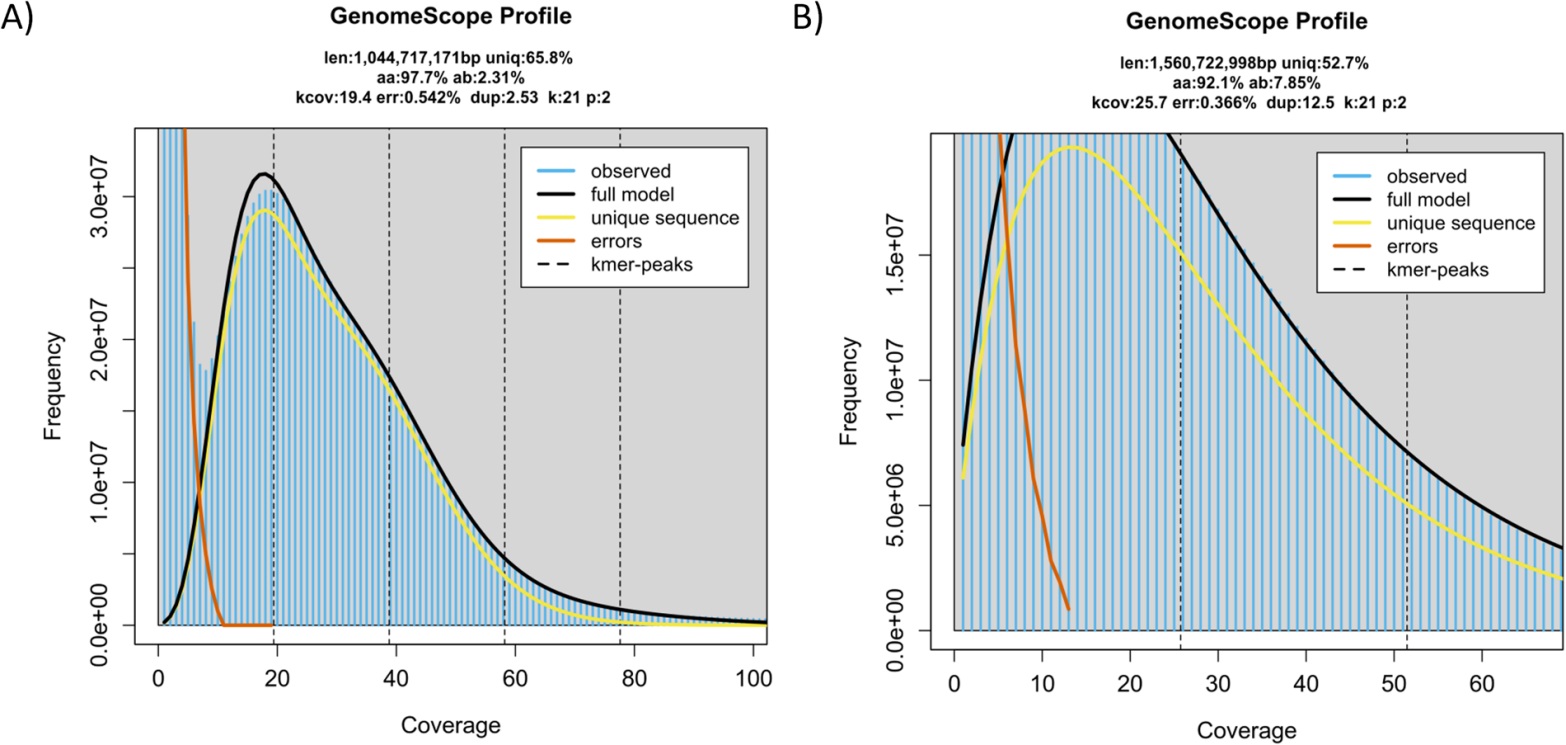
GenomeScope plots with the estimated genome size (*K*-mer = 21) of *A. flexuosa* (**A**) and *M. petechialis* (**B**).

**Fig. 5:**
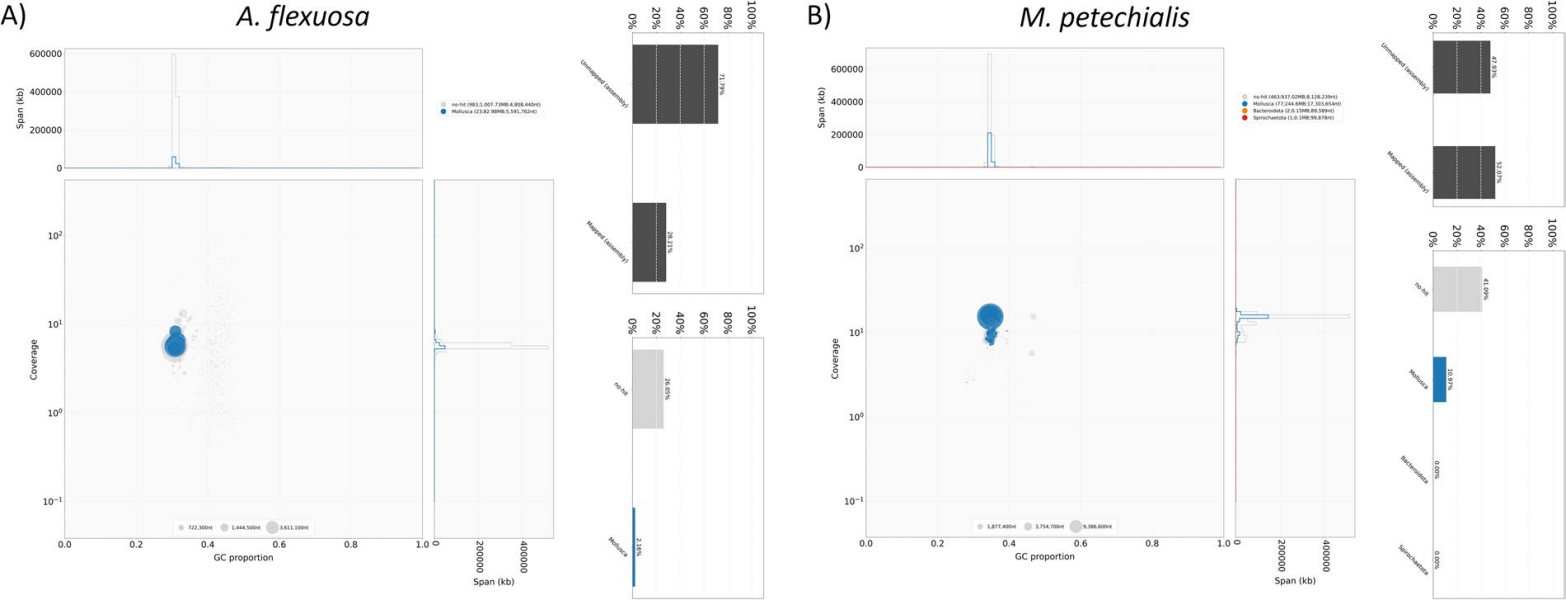
GC-coverage plots from BlobTools for genome assembly quality control and contaminants detection for *A. flexuosa* (**A**) and *M. petechialis* (**B**). The size of circles are proportional to the scaffold length. The upper bar plot shows the proportion of mapped and unmapped assembly to the NT database whereas the lower bar plot illustrates the distribution of the mapped assembly that are assigned to specific taxa.

The pseudochromosomes of the final assemblies were validated by inspecting the Omni-C contact maps using Juicer tools (version 1.22.01) (Durand *et al*.)^8^. Briefly, Omni-C reads were mapped and aligned by BWA with parameters “mem -5SP -T0”, the parsing module of the pairtools pipeline was used to find ligation junctions with parameters “--min-mapq 40--walks-policy 5unique--max-inter-align-gap 30--nproc-in 8--nproc-out 8”. The parsed pairs were then sorted using pairtools sort with default parameters, PCR duplicate pairs were removed using pairtools dedup with parameters “--nproc-in 8 --nproc-out 8 --mark-dups”, the pairs file was split using pairtools split with default parameters and used to generate the contact matrix using juicertools and Juicebox (v1.11.08)^8^. Regarding the genome characteristics of the assembly, the k-mer count and histogram were generated at k = 21 from Omni-C reads using Jellyfish (v2.3.0) (Marçais & Kingsford)^31^ with the parameters “count -C -m 21 -s 1000000000 -t 10”, and the reads.histo was uploaded to GenomeScope to estimate genome heterozygosity, repeat content and size using default parameters (v2.0) (http://qb.cshl.edu/genomescope/genomescope2.0/) (Ranallo-Benavidez *et al*.)^32^. The resulting GenomeScope plots can be found in Fig. 4. Omni-C reads and PacBio HiFi reads were used to measure assembly completeness and consensus quality (QV) using Merqury (v1.3)^33^ with kmer 20, resulting in 75.3568% and 96.1486% kmer completeness for the Omni-C data and 57.149 and 62.1256 QV scores for the HiFi reads, corresponding to 99.999% and 99.9999% accuracy for *A. flexuosa* and *M. petechialis*, respectively. In addition, BlobTools (v1.1.1) assigned most of the assembled scaffolds that mapped to the NT database to the taxon Mollusca and scaffolds that assigned to other taxa such as Bacteroidota were removed (Laetsch & Blaxter)^5^ (Fig. 5).

## Data Availability

No specific script was used in this work.
